# Telomere Length Variation in Juvenile Acute Myocardial Infarction

**DOI:** 10.1371/journal.pone.0049206

**Published:** 2012-11-07

**Authors:** Alessia Russo, Luigi Palumbo, Cristina Fornengo, Cornelia Di Gaetano, Fulvio Ricceri, Simonetta Guarrera, Rossana Critelli, Matteo Anselmino, Alberto Piazza, Fiorenzo Gaita, Serena Bergerone, Giuseppe Matullo

**Affiliations:** 1 Department of Genetics, Biology and Biochemistry, University of Turin, Turin, Italy; 2 HuGeF, Human Genetics Foundation, Turin, Italy; 3 Department of Internal Medicine, University of Turin, Cardiology Division, San Giovanni Battista Hospital, Turin, Italy; Sapienza University of Rome, Italy

## Abstract

Leukocyte telomere length (LTL) provides a potential marker of biological age, closely related to the endothelial dysfunction and consequently to the atherosclerotic process. To investigate the relationship between the LTL and the risk of premature acute myocardial infarction and to evaluate the predictive value of LTL on the onset of major cardiovascular events, 199 patients from 18 to 48 years old with first diagnosis of acute myocardial infarction were enrolled and were matched with 190 controls for sex and age (±1 year). Clinical data and coronary artery disease were evaluated at enrollment and at follow up. LTL was measured at enrollment using a quantitative PCR-based method. No significant differences were observed in LTL between cases and controls (p = 0.20) and with the presence of coronary artery disease in patients (p = 0.47). Hypercholesterolemic cases presented LTL significantly longer than cases without hypercholesterolemia (t/s: 0.82±0.16 p = 0.79 and t/s norm: 0.79±0.19 p = 0.01), as confirmed in multivariate regression analysis (p = 0.005, β = 0.09). Furthermore, multivariate regression analysis showed LTL significantly shorter in hypertensive cases than in normotensive cases (p = 0.04, β = −0.07). One hundred seventy-one cases (86%) ended the average follow up of 9±5 years, 92 (54%) presented a major cardiovascular event. At multivariate regression analysis the LTL detected at enrollment did not represent a predictive factor of major cardiovascular events nor it significantly impacted with cumulative events. Based on present cohort of young Italian patients, the LTL did not represent a marker of acute myocardial infarction nor had a predictive role at medium term follow up.

## Introduction

Coronary heart disease (CHD) is an age related disease, and aging is considered a major risk factor of atherosclerosis [1]. Ischemic heart disease occurring in young adults is an uncommon finding, representing from 4 to 10% of the total case population [2], and it is associated in the majority of cases with a premature coronary artery disease (CAD) [3]. Even if the juvenile CHD is less frequent, it is a major health burden with an unfavorable prognosis at medium-long term [4].

Recent evidence [5] support a close relationship between the biological age and atherosclerotic process indicating that the mechanisms involved in cellular senescence might have a role in endothelial dysfunction. According to this, it should be remarkable to find a marker of cellular biological age able to predict the risk of early onset of atherosclerosis and consequently of a premature CHD. An increasing interest is focused on telomere, tandem repeat DNA sequences situated at the ending part of the eukaryotic chromosomes [6], that progressively shorten during the cell divisions critically influencing the senescence and programmed cell death. The telomere length represents therefore a sort of biological cell clock and shorter telomeres reflect a more advanced biological age [7]. Short telomeres, in fact, have been detected in senescent endothelial cells present in atherosclerotic plaques [5], [8] and telomeres are remarkably shorter in patients with aging associated disease such coronary artery disease and chronic heart failure [9]. Nevertheless, the studies published so far investigated the relationship between mean telomere length and the cardiovascular disease only in middle age subjects [10], [11] and at present few studies are suitable to determine the actual role of telomere length in the beginning of acute myocardial infarction in young adults.

The aim of the present case-control study is to evaluate the relationship between the leukocyte telomere length (LTL) and the risk of premature acute myocardial infarction in a cohort of Italian patients aged ≤48 years old. Moreover, we prospectively evaluated the patients during a medium term follow up to assess the prognostic impact of LTL with the onset of major acute cardiovascular event (MACE).

## Methods

### Participants

The case-control study enrolled 199 patients with first diagnosis of acute myocardial infarction (AMI) at ≤48 years old consecutively admitted in our Coronary Care Unit from March 1992 to May 2009 and 190 controls, frequency matched for age and sex. The AMI was diagnosed according to criteria of the Joint European Society of Cardiology/American College of Cardiology Committee [12]. All the subjects enrolled had Italian nationality. All controls selected were free of previously diagnosed cardiovascular disease or cancer. All subjects were interviewed with a standardized questionnaire concerning the following cardiovascular risk factors: smoking habits, hypertension, diabetes, hypercholesterolemia, body mass index (BMI), family history of CAD. All definitions were based on those recommended in the European guidelines on cardiovascular disease prevention in clinical practice issued 1998 [13]. In patients the following measurements were also collected: electrocardiographic presentation at AMI (with ST segment elevation myocardial infarction, STEMI or without ST segment elevation myocardial infarction, NSTEMI); left ventricular ejection fraction (LVEF) and coronary vessel disease assessed by coronary angiography during the hospitalization.

Follow-up ended in April 2010 with an outpatient visit or, if not possible, a telephone interview. All patients answered to the same standardized questionnaire completed at baseline. Cardiovascular events were defined as cardiac death, recurrent AMI, heart failure needing hospitalization, stroke and angina pectoris needing a revascularization procedure [14]. Dead patients were assessed during follow-up also through demographic and hospital records.

The patients were enrolled after giving written informed consent concerning the study. This study was performed according to the principles of the Declaration of Helsinki and in agreement with ethical requirements. An internal ethical review board at HuGeF (Human Genetics Foundation Ethics committee/17-11-2011) approved the study. No sex-based or ethnic-based differences were present; most of the subjects are of Caucasian origins.

### Measurement of Leukocyte Telomere Length

All subjects enrolled underwent a venous blood sample drawn. Leukocyte DNA was extracted by the salting-out method. DNA concentration was measured with NANODROP 8000 (ThermoScientific) and all samples have been diluted to a fixed concentration of 2 ng/µl.

The total amount of telomeric DNA was measured by Real-Time Quantitative PCR, by measuring for each sample the relative amount of telomeric DNA (t) as compared to the amount of a single copy gene DNA (s) in the same sample (t/s ratio).

Real Time PCR telomeric DNA measurement and single gene DNA measurement were performed in separate plates due to different annealing temperatures of the specific primers.

To guarantee comparability between plates, a commercial DNA (Human DNA Male, Applied Biosystems) used as calibrator was plated in each PCR run.

A second commercial DNA (Human DNA Male, Applied Biosystems) was diluted serially to produce a 5-point standard curve (ranging 20 to 1.25 ng DNA) and was included in each plate to measure the telomeric and single copy gene DNA amounts.

The gene *36B4*, which encodes the acidic ribosomal phosphoprotein P0, was used as single copy reference gene as described in Cawthon [15].

Primers sequences [35] and annealing temperatures were as follows:

TEL 1 - 5′ CGG TTT GTT TGG GTT TGG GTT TGG GTT TGG GTT TGG GTT - 3′.

TEL 2 - 5′ GGC TTG CCT TAC CCT TAC CCT TAC CCT TAC CCT TAC CCT - 3′.

36B4 1- 5′ CAG CAA GTG GGA AGG TGT AAT CC - 3′.

36B4 2 - 5′ CCC ATT CTA TCA TCA ACG GGT ACA A - 3′.

A total 15 µl of PCR mix containing 1X SYBR Green (Power SYBR® Green PCR Master Mix, Applied Biosystems), 0.2 pmol/µl each primer and 4 ng genomic DNA was prepared for each sample. Each sample as well as calibrator sample and standard curve points were run in triplicate.

Cycling conditions were as follows: 95°C × 7 m [95°C × 15 s, 54°C × 2 m]×30 for PCR amplification of telomeric regions; 95°C × 7 m [95°C × 15 s, 58°C × 1 m]×40 for PCR amplification of *36B4* gene.

To check for PCR reaction specificity, a melting analysis step was included at the end of each PCR run: no non-specific PCR product was detected.

A 7900HT Fast Real-Time PCR System (Applied Biosystems) with SDS Software version 2.3 (Applied Biosystems) was used. All PCR runs were inspected by a trained operator, and the threshold value suitable for the analysis of all the PCR batches was chosen.

The t/s of all the samples has been compared with the t/s of the same commercial DNA (Human DNA Male, Applied Biosystems) in order to calculate a t/s normalized ratio (t/s norm) for each sample.

The commercial DNA included in all the plates showed low variability (coefficient of variation _36B4_Ct_CTR_ = 1.46%; coefficient of variation _TEL_Ct_CTR_ = 1.36%) in Threshold Cycle (Ct) values among different PCR batches (n = 5), confirming a good repeatability of the assay.

In our experimental conditions the standard curves’ correlation coefficients were all R^2^>0.99.

Leukocyte telomere length from 30 healthy individuals (age range 22–61) were measured by Real-Time Quantitative PCR and compared with relative mean TRF lengths (manuscript in preparation) in the same samples measured by traditional Southern blot methods [36]. The Pearson correlation coefficient between these two different approaches to measuring telomeres was 0.6 (p<0.001). The same 30 samples were run in two different times to assess measurement variability and the Pearson correlation coefficient was 0.7 (p<0.0001).

### Statistical Methods

We tested for the differences between two groups (case-control status, above and below the median of normalized t/s ratio, and other dichotomic categorical variables ) using the non-parametric Wilcoxon sum rank test.

We performed multivariate regression analysis using a general linear model (case-control status as dependent variable) including all the variables of interest (hypertension, hypercholesterolemia, diabetes, familiar history of CAD, smoking status and BMI) and adjusting for age and sex as covariates. The probability of survival to the different outcome events over time for each group was calculated according to the Kaplan-Meier method and compared by log-rank test. All the analyses have been done for t/s ratio and for normalized t/s ratio, however, data are presented only for the latter. Data were analysed using SAS V 9.2 and STATA v 10.0.

## Results

The baseline characteristics and the main cardiovascular risk factors are shown in Table 1. Eighty-nine percent of patients were males. The main cardiovascular risk factors recorded in patients were the smoking (82% vs 40%, p<10^−4^), the family history for CAD (61% vs 17%, p<10^−4^) and the hypertension (38% vs 7%, p<10^−4^). Moreover, the cases were most frequently hypercholesterolemic respect to the controls (61% vs 31%, p<10^−4^), with a higher prevalence of diabetic subjects among cases (13% vs 2%, p<10^−4^). The majority of patients (69.8%, 139 patients) presented a STEMI and the LVEF was in general preserved (LVEF: 54%±10.5%). The mean LTL in cases was not statistically different from the controls (t/s norm: 0.77±0.19 vs 0.76±0.19 p = 0.20, from Wilcoxon sum rank test). There was no correlation between LTL and age, neither in cases (t/s norm, r = −0.001, p = n.s.) nor in controls (t/s norm, r = −0.001, p = n.s.). Concerning the cardiovascular risk factors, we did not find a significant association between hypertension and LTL in either cases (t/s norm, p = 0.06) and controls (t/s norm, p = 0.8) using the Wilcoxon sum rank test (Table 2), however, multivariate regression analysis showed LTL significantly shorter in hypertensive cases (p = 0.04, β = −0.07). Furthermore, hypercholesterolemic cases had LTL significantly longer than cases without hypercholesterolemia (t/s norm:0.79±0.19 p = 0.01) (Table 2) as confirmed at multivariate regression analysis (p = 0.005, β = 0.09). The lack of significant associations between hypercholesterolemia as well as hypertension and LTL in controls can be due to the lower frequency of these risk factors among them. No significant relationship (Table 2) was found between LTL and family history of CAD, (t/s norm, p = 0.64) in cases. Among controls, subjects with family history of CAD had LTL longer than controls without family history (t/s norm: 0.88±0.14 p = 0.002) (Table 3), data confirmed at linear regression analysis (p = 0.03). Regarding the analysis of LTL vs the presence of coronary artery disease, our data did not reveal any statistically significant correlation (t/s norm, p = 0.46) (Table 4).

**Table 1:** Baseline characteristics of 199 cases and 190 controls at enrollment.

Characteristics		Cases N (%)	Controls N (%)	P-value
Males		178 (89.4)	173 (91.1)	ns
Age		40.1±5	39.8±5	ns
Smoking	Yes	155 (82)	65 (39.6)	<10^−4^
	No	34 (18)	99 (60.4)	
Family history of CAD	Yes	120 (61.2)	14 (16.5)	<10^−4^
	No	76 (38.8)	71 (83.5)	
Hypertension	Yes	74 (37.8)	12 (7.3)	<10^−4^
	No	122 (62.2)	152 (92.7)	
Hypercholesterolemia	Yes	116 (61)	49 (30.6)	<10^−4^
	No	75 (39.3)	111 (69.4)	
Total cholesterol		214.4±48	207.8±36	ns
Triglycerides		156.5±112	107.4±61	<10^−4^
LDL		136.6±105	125.4±38	ns
HDL		49.8±18	57.6±14	<10^−4^
Diabetes	Yes	26 (13.3)	3 (1.8)	<10^−4^
	No	170 (86.7)	161 (98.2)	
BMI	Yes	105 (60.3)	64 (45.1)	0.007
	No	69 (39.7)	78 (54.9)	
BMI		26.5±5	24.9±3	10^−4^

Data are shown as percentages for categorical variables and means for continuous variables. CAD, coronary artery disease; BMI, body mass index; LDL, low density lipoprotein; HDL, high density lipoprotein.

Active smokers were included in the group of smokers whereas subjects who have never smoked or who have stopped smoking for ten years or more were considered as non-smokers.

Hypertensive participants were defined by systemic blood pressure ≥140/90 mmHg or treatment with antihypertensive therapy.

Hypercholesterolemia was determined by total cholesterol >220 mg/dl.

Obesity was defined by BMI >25 kg/m^2^.

Diabete was determined by medical history or fasting blood glucose >126 mg/dl on two testing occasions or glucose overload test.

**Table 2:** Wilcoxon sum rank test on leukocyte telomere length presented t/s ratio normalized and cardiovascular risk factors of cases (n = 199).

Variable	LTL (t/s norm)	p value
Male sex (M vs F)	0.77±0.19 vs 0.71±0.19	0.20
Smoking (Ever vs Never)	0.76±0.20 vs 0.80±0.17	0.45
Hypertension (Yes vs No)	0.74±0.18 vs 0.79±0.20	0.06
Hypercholesterolemia (Yes vs No)	0.79±0.19 vs 0.72±0.19	**0.01**
Diabetes (Yes vs No)	0.80±0.17 vs 0.76±0.20	0.47
Family history of CAD (Yes vs No)	0.78±0.19 vs 0.76±0.20	0.64

Data are presented as means and standard deviation. LTL, leukocyte telomere length; CAD, coronary artery disease.

**Table 3:** Wilcoxon sum rank test on leukocyte telomere length presented t/s ratio normalized and cardiovascular risk factors of controls (n = 190).

Variable	LTL (t/s norm)	p value
Male sex (M vs F)	0.80±0.18 vs 0.76±0.20	0.36
Smoking (Ever vs Never)	0.84±0.19 vs 0.80±0.18	0.28
Hypertension (Yes vs No)	0.81±0.12 vs 0.82±0.19	0.78
Hypercholesterolemia (Yes vs No)	0.81±0.17 vs 0.82±0.19	0.92
Diabetes (Yes vs No)	0.91±0.04 vs 0.82±0.18	0.29
Family history of CAD (Yes vs No)	0.88±0.14 vs 0.71±0.18	**0.002**

Data are presented as means and standard deviation. LTL, leukocyte telomere length; CAD, coronary artery disease.

**Table 4:** Wilcoxon sum rank test on leukocyte telomere length presented t/s ratio normalized and coronary artery disease in patients.

	LTL (t/s norm)	p value
Normal coronary artery (n = 22) vs coronary artery disease (n = 170)	0.81±0.14 vs 0.77±0.20	0.46
Monovessel disease (n = 96) vs Multivessel disease (n = 72)	0.75±0.20 vs 0.79±0.19	0.22

Data are presented as means and standard deviation. LTL, leukocyte telomere length.

**Figure 1:**
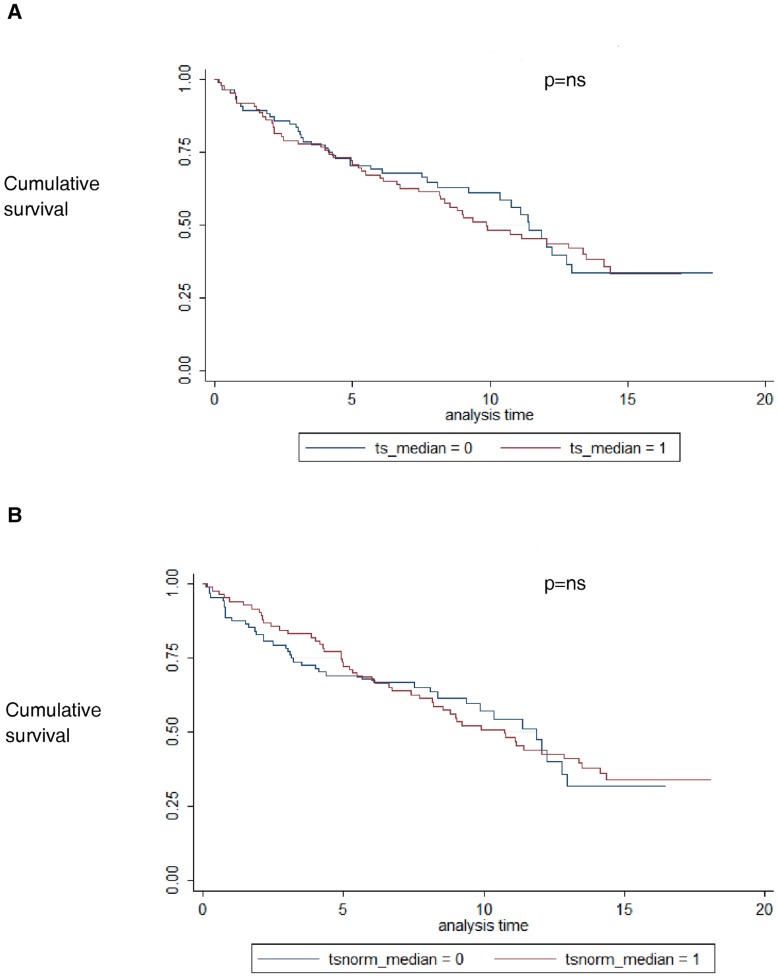
A, Event free survival from major cardiovascular events in patients on basis of leukocyte telomere length at enrollment measured as t/s. B, Event free survival from major cardiovascular events in patients on basis of leukocyte telomere length at enrollment measured as t/s normalized.

About 86% of patients enrolled (171 cases) completed the follow-up. During a medium period of 9±5 years 92 out of 171 (54%) remaining patients presented a MACE. AMI occurred in 37 patients, a percutaneous coronary intervention due to angina was performed in 35 patients and 17 died for cardiac death. Only 1 case presented a stroke and 2 patients were hospitalized for heart failure. The LTL detected at enrollment did not significantly impact with the composite CVD end-point (Figure 1A and Figure 1B), nevertheless, as shown in Figure 1B, cases with shorter LTL seemed to have a worse prognosis considering the first five-year after AMI, although the difference was not statistically significant.

## Discussion

In this Italian prospective case-control study, we did not show neither a significant association between the LTL and the AMI occurred in young adults nor a relationship between the LTL and the onset of MACE during a medium term follow up.

In contrast with our results, Maubaret *et al.* [11] in HIFMECH (hypercoagulability and impaired fibrinolytic function mechanisms predisposing to myocardial infarction) study have shown a significant relationship between LTL and incidence of AMI in patients <60 years, suggesting that shorter LTL could be a marker of coronary heart disease in a later age. Same results were reported by Brouilette *et al.* [16] in a case control study conducted in English patients with AMI at 50 years or less and by the same author in West of Scotland Primary Prevention Study (WOSCOPS study) [10] in middle aged men (45–64 years). Finally Zee *et al.* observed that mean LTL was shorter in subjects who developed AMI than in subjects who did not [17].

Our study is the first to investigate the LTL in a well-selected Italian survey and our data do not reveal a significant difference in LTL between case and controls. However, the enrolled patients in Brouilette *et al.* [10], [16] and in Zee *et al.* [17] were in average older than our patients, not a trivial aspect, considering that the telomere length decreases progressively with age and in the younger patients the expected major activity of DNA repair systems could determine a minor telomere shortening.

Our cohort is similar for risk profile to the juvenile populations investigated by previous studies [2], [18], [19] and in particular our patients are representative of an Italian survey of juvenile AMI [20] being the majority of cases smokers, hypercholesterolemic, hypertensive and presenting a family history for CAD.

Regarding a possible correlation between the LTL and a family history of CAD, Salpea *et al.* [21] demonstrated in the European Atherosclerosis Research Study II (EARSII) a shorter LTL in young male students with a paternal history of premature myocardial infarction with respect to the age-matched controls with no family history of CAD. We found a longer LTL in controls with family history of CAD respect to the controls without family history (t/s norm: 0.88±0.14 p = 0.002), data confirmed at linear regression analysis (p = 0.03), whereas in cases with positive family history the LTL was slightly shorter respect to the cases with no history of CAD, although the difference was not statistically significant (p = 0.10).

Although the discordant results can be due to the limited number of subjects with family history in our study sample, mean age and health status could partly explain the differences with the study of Salpea *et al*. (2009) [21].

Some evidence suggest that telomere shortening may play a role in pathogenesis of hypertension [22] probably inducing genomic instability related to an early cellular senescence, as reported also by Giannotti *et al.* [23] who described a shorter telomere length in progenitor endothelial cells of prehypertensive and hypertensive patients. In a recent study Yang *et al.* [24] found a significant correlation between shorter LTL and hypertensive subjects in a Chinese population [25]. Moreover, in previous studies an inverse correlation was observed between pulse pressure [26], [27], pulse wave velocity [26] and LTL. Our data showed no significant association between hypertension and LTL neither in cases nor in controls using the Wilcoxon sum rank test, nevertheless hypertensive cases presented a shorter telomere length after correction for other risk factors (p = 0.04) at linear regression analysis. As already described, our cases are on average younger than patients considered in the above mentioned studies on hypertension, which is more clearly related to ageing than other risk factors (e.g., hypercholesterolemia).

We also found a significant association between hypercholesterolemia and LTL in cases, with longer LTL in cases with hypercholesterolemia. When we examined the correlation of total cholesterol, HDL and LDL with LTL, we found that HDL positively correlated with LTL with borderline significances in cases and controls together (t/s norm ρ = 0.1, p = 0.069). These results are consistent with a recent study in which total cholesterol was positively associated with LTL [28], as well as reduced HDL levels have been described associated with shorter LTL in healthy young adults [29]. According to Ramin Farzaneh-Far *et al.* [30], it is possible that individuals with shorter telomeres in proximity of a critical threshold, can reactivate their enzyme telomerase (usually active at low levels in peripheral blood leukocytes) leading to a feedback regulation responsible for the increasing of LTL.

Previous studies [5], [8] showed an association with shorter telomere length and atherosclerosis revealing that endothelial cell senescence induced by telomere shortening may contribute to atherogenesis but few studies investigated directly the association between mean LTL and the coronary artery disease. Mainous *et al.* [31] reported in a cohort of 325 patients aged 40–64 years free of previously diagnosed CVD an inverse correlation between the telomere length and the coronary artery calcification, whereas De Meyer *et al.* [32] in the Asklepios study demonstrated that LTL is not an independent determinant of atheromatous plaques present in patients free from established CVD. In our limited sample, we did not show any relationship between LTL and coronary artery disease, the telomere length in fact did not differ significantly in cases with normal coronary at angiography respect patients with a vessel disease. Although Wilson *et al.* [33] showed that the leukocyte telomere DNA content predicts vascular telomere DNA content, the leukocyte telomere length still represents a surrogate marker of the endothelial telomere length. In fact, one of the main limitations of this study, as of many other above mentioned studies, is that we are measuring TL in leukocytes.

Moreover, studies with longer follow-up are necessary to better understand the role of LTL in coronary artery disease.

During a median follow up of 9±5 years, the majority of patients remained in the study at the last follow up, 54% of these presented a MACE. Our data show an unfavorable prognosis at medium term, confirming the results of Fournier *et al*. [4] who reported an event-free survival rates at 10 years of 60%, in a cohort of patients affected by AMI at ≤40 years old and of Fornengo *et al.* [34] who found a poor five-year prognosis of juvenile AMI with one in every five patients presenting a new cardiovascular event.

The measurement of LTL at enrollment did not result to be an independent predictor of any MACE at follow up nor it significantly impacted with cumulative survival, describing a lack of association with coronary artery disease in young patients (≤48 years old).

Finally, we did not find a correlation between age and telomere length probably due to the narrow age range (18–48 years) of our population, which is probably the most flattened area of the LTL-age curve. This result is in agreement with the study of Jeanclos *et al.* [27] in which subjects were 18 to 44 years old and with Salpea *et al.* [21] who studied subjects ranging 18 to 28 years old. It appears, moreover, that different phases in the rate of telomere attrition exist throughout life. The initial phase (i.e., birth to 5 years) is marked by a relatively high rate of telomere attrition. The subsequent phase that includes adolescence and young adulthood is marked by an apparent stabilization of telomere length. Thereafter, telomere attrition resumes at a slower rate than during the first 5 years of life [37]. The majority of subjects we studied were within the age range in which the rate of telomere attrition slows down or levels off altogether, accounting for the lack of correlation between the telomere length and age in this group.

To summarize, in this Italian cohort of patients aged ≤48 years old, LTL did not represent neither a marker of premature myocardial infarction nor a predictor of MACE at medium term follow up. Although current data revealed that the LTL is a marker of biological aging and, side by side, of the cardiovascular disease, further studies are mandatory to evaluate the effective roles of leukocyte telomere length and the use as biomarker for coronary heart disease occurring in young patients.
